# Test Methods for Characterizing the Properties of Fiber-Reinforced Polymer Composites at Elevated Temperatures

**DOI:** 10.3390/polym14091734

**Published:** 2022-04-24

**Authors:** Venkatesh Kodur, Svetha Venkatachari, Vasant A. Matsagar, Shamsher Bahadur Singh

**Affiliations:** 1 Department of Civil and Environmental Engineering, Michigan State University (MSU), 3546 Engineering Building, East Lansing, MI 48823, USA; venkat28@msu.edu; 2 Department of Civil Engineering, Indian Institute of Technology (IIT) Delhi, Hauz Khas, New Delhi 110 016, India; matsagar@civil.iitd.ac.in; 3 Department of Civil Engineering, Birla Institute of Technology & Science (BITS) Pilani, Vidya Vihar, Pilani 333 031, India; sbsingh@pilani.bits-pilani.ac.in

**Keywords:** natural fiber composites, bio-based FRP, fire performance, thermal properties, mechanical properties, standardized test methods

## Abstract

Recent research trends focus on developing bio-based (derived from agricultural byproducts) fiber-reinforced polymer (FRP) composites for structural applications. Fire resistance is one of the key issues that need to be addressed for the use of these FRP materials in buildings. The thermal and mechanical properties of the constituent materials essentially determine the fire performance (and the fire resistance rating) of a structural member, and these properties vary with temperature. Further, the properties of composite materials such as the FRP are highly influenced by the composition and type of fibers and matrix, and these thermo-mechanical properties also vary significantly with temperature. Due to this variation, the fire resistance of FRP materials (both conventional and bio-based) poses a major concern for use in buildings. Currently, very few standardized test procedures are available for evaluating the high-temperature material properties of FRP composites. In this paper, a review of testing protocols and procedures for undertaking tests on FRP materials at various elevated temperatures for evaluating their properties is carried out. Recommendations are provided on the most suitable test methods, specimen conditions, testing regime, and other issues associated with testing at elevated temperatures. In addition, the applicability of the proposed test methods is illustrated through a case study on conventional FRP specimens. Further, the applicability of the recommended test procedures for measuring high-temperature properties of bio-based FRP composites is highlighted.

## 1. Introduction

Over the last three decades, fiber-reinforced polymer (FRP) composites are increasingly used in civil engineering applications due to their superior properties such as high strength and stiffness-to-weight ratios, resistance to corrosion, low maintenance, and design flexibility compared to conventional construction materials such as steel and concrete [1]. Currently, these FRPs are used in structural applications as internal reinforcement in concrete members or as external reinforcement in the repair, retrofit, and rehabilitation of concrete structures. In addition, panels made of FRP composites are used as non-structural members on walls of buildings and decks in bridges. Conventional FRP composites comprise synthetic fibers such as glass, carbon, and aramid that are derived from petroleum-based products. However, the emerging trend in the construction sector is to reduce carbon emissions due to growing environmental concerns about the high CO_2_ emissions resulting from the wider use of petroleum-based products.

Natural fibers are being considered as a potential alternative, in place of synthetic fibers, for fabricating bio-based composite materials. Biomass converted to ceramic nanoparticle in forced cellulosic fibers and combined with agricultural side products (rice, corn husk, etc.) is being processed to prepare the bio-based FRP composites. Typically, plant-based fibers are derived from agricultural waste/stubble, and this alternative is gaining considerable interest owing to their superior advantages over synthetic fiber, such as their relatively low weight, locally available raw ingredients, low cost, eco-friendly, and acceptable strength and stiffness properties. Such bio-based FRPs can be utilized in buildings in the form of fibers, rebars, strips, sandwich panels, and other decorative face sheets to achieve energy savings and economy in the construction sector. In addition, the overall carbon footprint resulting from the construction sector can be reduced.

Some of the major issues with the FRP composites, including bio-based FRPs that limit their application in buildings, are their high flammability and poor fire resistance properties. The FRP components are highly combustible and decompose even at low to moderate temperatures in a fire situation releasing soot, smoke, and toxic gases. This leads to the generation of toxic gases and poor visibility conditions, which can hinder the occupant evacuation process and firefighting operations in a building. From a structural perspective, the FRP composites rapidly lose strength and stiffness at moderate temperatures of 200 °C to 300 °C due to the degradation in the properties of the polymer matrix with temperature rise [1]. Moreover, the bond between the FRP reinforcement and concrete degrades significantly, especially at temperatures beyond the glass transition temperature (*T*_g_), which affects the force transfer between the structural member and the FRP reinforcement [2]. The above-discussed temperature range is for conventional FRP composites. There are hardly any data available on property variation in bio-based FRP composites.

Structural members in buildings are required to meet the fire resistance ratings prescribed codes and standards, in addition to other criteria such as flame spread ratings, smoke generation classifications, etc. For evaluating the fire resistance of an FRP-strengthened concrete structural member, information regarding the thermal and mechanical properties of the FRP composites together with that of concrete and steel reinforcement are needed. The thermal properties determine the thermal behavior of the composite material (and structural member) including the extent of temperature rise in a member. Mechanical properties, which include strength, modulus, creep, and bond strength, determine the loss of structural capacity, and the extent of deformation in a member with fire exposure time. The properties of the FRP vary significantly with temperature compared to that of conventional construction materials and need to be reliably measured through standardized test methods.

The material properties of conventional FRP have been studied widely at ambient temperature conditions [2,3,4]. In the last two decades, there have been numerous test programs implemented to obtain high-temperature property data for conventional FRP composites and thus various researchers have used different test methods to evaluate these properties. High-temperature property relations have also been proposed for some of the commonly used conventional FRP composites in the past literature [5]. However, there are limited property data both at ambient and elevated temperatures for the bio-based FRP [6,7], and this is due to the lack of standardized test procedures and limited research effort in this direction. While there is some guidance on needed test methods for characterizing room temperature properties of the FRP composites, there is not much guidance available on the test methods for property characterization at elevated temperatures. For this purpose, a comprehensive review of test methods and procedures specified in some of the current standards (including the American Society for Testing and Materials (ASTM) and the International Organization for Standardization (ISO) standards) and published papers is carried out. Based on this detailed review, the most applicable test methods for high-temperature material characterization of the FRP composites, including those of bio-based FRPs, in the temperature range of 20 to 600 °C are presented in this paper.

## 2. Recent Advances in Bio-Based FRP Composites

As mentioned earlier, there are many merits to synthetic fiber-based composites with certain disadvantages too, such as difficulty associated with the disposal of synthetic fibers and high cost. Currently, researchers are focusing on natural fiber-reinforced composites for building applications in the fabrication of structural and non-structural elements, and various other infrastructure systems especially where sustainable materials of low weight and moderate strength are required. For the past few decades, natural fiber composites have been used in the automobile industry for making various body parts, rotor blades, and lining materials. In the infrastructure market, bio-based FRPs are utilized in making water tanks, load-carrying beams and columns, pedestrian footpaths, and panels for multi-purpose use [8].

Natural fibers are also used to improve the performance of the composite through hybridization with synthetic fibers. Such hybridization reduces moisture absorption, in addition to balancing the cost of the materials and allowing for surface modification using chemicals [9]. The hybrid composites can be manufactured using only one type of natural fibers or with various combinations. The composites made using different types of natural fibers have shown to exhibit significant advantages in applications over various sectors. The natural fiber-based hybrid composites have reduced the cost of the materials along with a significant reduction in the weight of composite parts [10]. Furthermore, the natural–natural fiber type of hybridization has also improved the impact strength of the composite [11].

Natural fiber-based sandwich composites are also being used in the fabrication of flexural members such as beams, bridge decks, and girders [12]. Ticoalu et al. [13] have provided a review of the recent advancements in natural fiber composites for structural and infrastructure applications. It must be also noted that the use of natural fibers in buildings and other non-vernacular architectural systems may lead to a significant reduction in energy consumption and greenhouse gases emission, which in turn reduces the carbon footprint and global warming. Steffens et al. [14] have given details of the use of natural fiber-based composites for architectural applications.

Currently, to prevent the deterioration of reinforced concrete structures due to aggressive environmental conditions, natural–synthetic fiber-based hybrid composites are also being used by wrapping these composites over the exposed surface of concrete structures. For example, the wrapping of hybrid sisal glass fiber-reinforced composite polymer (HSGFRP) around the concrete cylinder has improved the performance of the test specimen [15]. Furthermore, Beradi and Iannace [16] have reported that natural fibers can be used instead of synthetic fibers in sound-absorbing panels as natural fibers have good thermal insulation and sound absorption properties, and at the same time, these fibers do not pose any health hazards while manufacturing the parts. The high porosity of natural fiber-based composites allows for the absorption of sound waves and aids in soundproofing.

Thus, from the above literature, it is observed that natural fiber-based composites are used as a reinforcing material globally due to their inherent advantages and have a wide variety of applications in many sectors where synthetic fibers have been used so far. Most importantly, an attempt is made by researchers globally to extract the natural fibers from agricultural waste and use them to develop natural fiber-based fiber-reinforced polymers for different structural applications and, in turn, contribute to the growth of national gross domestic product (GDP) and generate the agro-based economy and its employment opportunities.

The material property data for bio-based FRP are limited to room temperature conditions and also mainly to certain mechanical properties of the FRP. Table 1 presents some of the room temperature material properties of natural fibers and their composites that are reported in the current literature [1,6,17,18,19]. The properties of conventional fibers and their composites are also included in Table 1 to illustrate the difference in their properties as compared to the bio-based FRPs.

As seen from Table 1, the natural fiber-based composites have lower strength and stiffness as compared to those of the conventional synthetic FRPs and thus, the bio-based FRP composites are more suited for building applications where the span of the structural members is small and the needed strengthening levels are low. In such building applications, fire safety is a critical consideration and structural members have to satisfy minimum fire resistance requirements. The key information needed for evaluating the fire resistance of structural members that incorporate bio-based FRP composites is the temperature-dependent thermal and mechanical properties of such FRP composites. For this purpose, this paper discusses the critical material properties of FRP composites that are of interest from the fire performance perspective and presents test methods and procedures to evaluate the thermal and mechanical properties at room and elevated temperature conditions.

## 3. Properties of Interest for Evaluating Fire Resistance

Temperature-dependent properties of FRP are essential in addition to other constituting materials for evaluating the fire performance of FRP structural members. The key properties are discussed below, in detail.

### 3.1. Flammability and Smoke Density Properties

Flammability and smoke density properties determine the flame spread and toxicity of a material when used in building applications. These properties do not have a direct relationship with the fire resistance (or structural response) of an FRP composite member. However, these properties are of importance from a fire safety point of view since a high rate of flame spread and toxic smoke development pose a severe hazard to the occupants of a building. For this reason, structural members in buildings must meet certain flame spread index (FSI) and smoke density index (SDI) requirements as prescribed in current codes. When the FRP is incorporated into a structural member, the FSI and SDI ratings are altered due to the combustible nature of the FRP. Unlike steel and concrete, which are non-combustible, the FRP is a combustible material and thus is flammable in a fire scenario. The material composition of the FRP composite largely determines the flame spread and generation of toxic smoke when exposed to fire.

### 3.2. Thermal Properties

The thermal properties of the FRP that are of interest from the perspective of fire performance of a structural member include thermal conductivity, specific heat, thermal expansion, and glass transition temperature (*T*_g_). The thermal conductivity and specific heat of the FRP composite have a direct influence on the rate of temperature rise within the structural member, and hence, on the fire resistance of an FRP composite member. The glass transition temperature indicates the temperature at which the polymer matrix turns to a viscous state leading to a drastic reduction in the strength and stiffness properties of the FRP composite, which in turn can severely impact the fire resistance (structural capacity) of an FRP composite member. Hence, the glass transition temperature has a direct relationship with the fire resistance of an FRP member. Finally, the thermal expansion of the FRP composite influences the stresses and deformation response of an FRP composite member, which in turn can directly affect the fire resistance of an FRP composite member. Depending on the type and amount of the FRP used, the level of influence that the thermal properties have on the fire response varies. For instance, in standalone FRP components and concrete members wrapped externally with the FRP laminates, the FRP composites are exposed to fire directly and thus, can affect the heat transfer within the member. In this case, the thermal properties of the FRP cannot be neglected in the fire resistance evaluation. However, when the FRP composite is used as an internal reinforcement within a concrete member, the thermal properties of the FRP tend to have negligible influence owing to the smaller cross-sectional area of the FRP reinforcement, as compared to that of the concrete section. In this case, the FRP reinforcement can be considered similar to that of steel rebar in the thermal analysis [20].

In addition, the thermal properties of the FRP composite can vary significantly with the material composition of the FRP, the orientation of the fibers, specimen conditions, testing conditions, and level of temperature rise. Further, the properties of the FRP composite are not isotropic, and these composite materials can exhibit different thermal properties in different directions. Very limited information is available on the variation in these properties with temperature and the available data are limited to conventional FRP composites at room temperature. For evaluating these properties for bio-based FRP, standardized tests are to be carried out from 20 °C to 300 °C. Beyond 300 °C the FRP tends to be unstable and starts to decompose [3].

The poor fire performance of the FRP composite is largely associated with the decomposition of the polymer matrix at high temperatures. At room temperature conditions, the polymer matrix maintains a glassy state. When temperatures increase to about 80 °C to 150 °C, the bonds of the polymer weaken, and the matrix turns into a viscous state when it reaches the glass transition temperature resulting in a significant drop in the strength and stiffness of the FRP material. For the conventional polymer matrix used in the FRP composites, the glass transition temperature is about 65–120 °C [21] but such temperatures are unavailable for many bio-based FRP composites. The glass transition temperature of FRP composites depends on the type and composition of the polymer matrix.

Standardized test methods are needed to minimize the variations in thermal property measurements. Table 2 presents the available test standards for thermal property evaluation and the temperature range of interest for the FRP composites.

### 3.3. Mechanical Properties

The mechanical properties of the FRP materials that influence the structural capacity and deformation response at elevated temperatures are tensile strength, elastic modulus, and stress–strain relations. In the conventional FRP, the embedded synthetic fibers can retain much of their strength up to 600 °C or higher. In the case of plant-based (natural) fibers, depending on the fiber composition, preliminary studies have shown that they are only able to retain a small part of their strength in the 180 °C to 400 °C range [7]. In both types, it is the polymer matrix of the FRP composite that is highly vulnerable to decomposition even at low to moderate temperatures of 65 to 140 °C [21]. Due to this, the FRP composite, as a whole, starts to lose strength and stiffness at temperatures beyond 100 °C. The degradation in properties of the FRP is highly dependent on the type of matrix and fiber, composition, the orientation of fibers, specimen conditions at the time of testing, and as well as the testing regime (such as the heating rate and loading) [5]. Moreover, the combustible nature of the FRP composites poses serious challenges and difficulties in undertaking high-temperature tests on these materials. Reliable characterization of the mechanical properties of the FRP requires standardized test procedures and methods.

Another mechanical property that significantly influences the fire response of the FRP-strengthened members is the bond strength. Sufficient bond strength is required between fibers and matrix and as well as the FRP composite and concrete to facilitate the transfer of stresses between the matrix and fibers and between the concrete member and the FRP system and vice-versa. However, due to the vulnerability of matrix performance to elevated temperatures, the bond properties of the FRP undergo significant degradation even at a moderate rise in temperature of 80 °C [1], which can affect the fire resistance of the member. Moreover, the type of FRP reinforcement, surface geometry, and glass transition temperature influence the extent of bond retained at the FRP-concrete interface at elevated temperatures [1]. Very limited data are available on the variation of bond strength with temperature rise, since measuring this property at high temperatures is highly challenging. Designing the grips to hold the specimen without crushing the anchor (or the end) zone of the member at high temperatures is critical apart from other testing parameters including the specimen size and conditions and the heating or loading rate. Proper design on anchors might also be critical while measuring the tensile and bond strengths of bio-based FRP to ensure that the failure of the specimen does not occur in the end zones during the test.

Yet another mechanical property that influences the fire response of the FRP components is the creep strain. In general, creep strain in the FRP increases with an increase in temperature and is largely governed by the type of polymer, type of fiber, and orientation of fibers in the FRP composite. Creep strains are extremely difficult to measure at elevated temperatures, and the degradation of the polymer matrix makes it even harder to measure this property of the FRP at high temperatures. Therefore, very limited information is available on the high-temperature creep strain of the conventional FRP. Since the FRP area is much smaller than that of concrete in the overall strengthened member, creep in the FRP is often neglected in fire resistance analysis [1].

The above-described mechanical properties have a direct relationship with the fire resistance of the FRP composite member. The higher the tensile strength, stiffness, and bond strength of the FRP composite, the higher the load-carrying capacity and lower the deformations in the member at elevated temperatures, and hence, the higher the fire resistance of the member. Table 3 summarizes the available test standards for mechanical property evaluation and the temperature range of interest for the FRP composites. In the absence of standardized test procedures at elevated temperatures, the recommended test method used by past researchers for measuring the mechanical property of FRP composites is provided.

## 4. Test Methods

The test methods, conditions, and procedures for measuring the various flammability, smoke density, thermal, and mechanical properties of FRP composites are discussed below.

### 4.1. Flammability and Smoke Density Properties

Standardized test methods are recommended in the ASTM and National Fire Protection Association (NFPA) standards to evaluate the flame spread and smoke generation of structural materials. The relative burning behavior of building materials can be evaluated by measuring the FSI and the SDI through procedures specified in ASTM E84 [22] and NFPA 255 [23] standards. The smoke concentration and surface flammability properties can be measured using the test methods provided in ASTM E662 [24] and ASTM E162 [25] respectively. The necessary equipment and instrumentation for carrying out flame spread (“Steiner tunnel test”) and smoke generation tests on construction materials are available in certain laboratories such as the Under Writer Laboratories [3]. Typically, the smoke generation and flame spread classifications are specified for FRP products, in material data sheets by their manufacturers in directories based on the standardized test results obtained from these specialized testing laboratories. Such flame spread and smoke generation classifications are not yet available for bio-based FRP materials. The same testing procedures as given in ASTM E662 and ASTM E162 might be used to evaluate the FSI and SDI of bio-based FRP materials upon ascertaining their applicability.

### 4.2. Thermal Properties

The thermal properties of an FRP composite can vary significantly based on the test methods, specimen conditions (preparation), and the type of instrument (or equipment) used to measure these properties. This high variation is because unlike steel and concrete there can be a high variation in the fiber-matrix composition and also the instability of these composites even at moderate temperatures (100–300 °C). For measuring thermal properties of construction materials at room temperature, different test methods are specified in the ASTM standards such as ASTM C177 [26], ASTM C1363 [27], ASTM E1269 [28], and ASTM E831 [29]. However, at elevated temperatures, there are limited standardized procedures available for undertaking thermal property tests. In many cases, researchers have applied the room temperature procedure to evaluate the properties at elevated temperature, by only considering the temperature rise, however without properly accounting for other critical factors including the specimen conditions and loading and heating rates [30]. In this paper, the test procedures applied for measuring the thermal properties of FRP that are published in the literature and standards such as the ASTM and ISO standards are reviewed, and the most suitable procedure that can be utilized for measuring thermal properties of the bio-based FRP at elevated temperatures is recommended.

#### 4.2.1. Thermal Conductivity

The procedure to measure the thermal conductivity of the conventional FRP composites at ambient conditions is given in ASTM C177 [26] and ASTM C1363 [27] standards. ASTM C177 [26] specifies a standard test for measuring the thermal conductivity of materials employing a guarded-hot-plate apparatus and is similar to the test specified in the ISO 8302 standard [31]. A temperature difference is established across a flat FRP specimen of a given thickness by exposing their parallel sides to the constant heat source from the hot-plate apparatus. The power required to maintain this temperature difference is used to calculate the thermal conductivity of the FRP specimen. The test procedure specified by the ASTM C1363 [27] standard uses a hot box apparatus to compute the thermal performance of building materials and works on a similar principle as the ASTM C177 [26] test procedure. The difference between the two methods is only concerning the size of specimens that can be tested in each case. The ASTM standards, however, do not guide on measuring the thermal conductivity of building materials beyond 80 °C.

**Figure 1:**
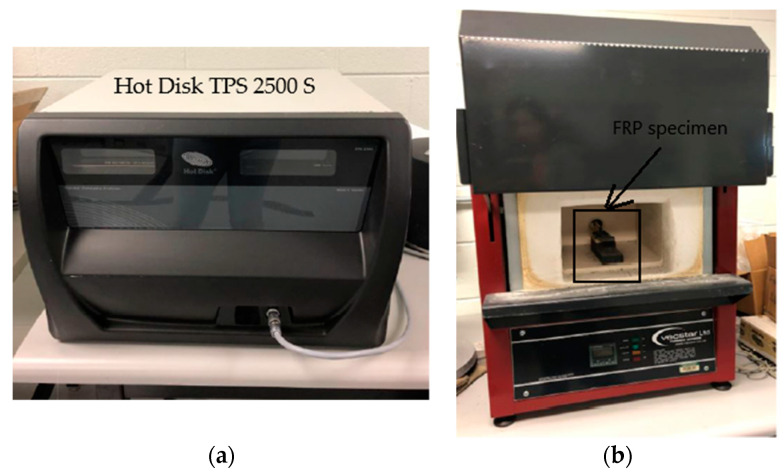
Equipment setup for hot disc method: (**a**) hot disc thermal constant analyzer and (**b**) furnace.

The ISO test standard 22007-2 [32] specifies a hot disc method for measuring thermal conductivity in the temperature range of 20 °C to 1000 °C. The thermal resistance experienced by a plane sensor fitted between two test specimens is measured using a thermal constant analyzer under transient heating conditions, which then provides the thermal conductivity of the specimen. Figure 1 shows the hot disc test setup using the Hot Disk TPS 2500 S test apparatus. Hot Disk TPS 2500 S is a commercially available thermal constant analyzer that conforms to the ISO 22007-2 standard [32]. The detailed procedure for measuring the thermal conductivity using the hot disc method can be found in [30].

From the different methods discussed above, it is recommended to use the hot disc method specified in ISO 22007-2 to evaluate the thermal conductivity of FRP specimens (conventional or bio-based FRP) in the temperature range of 20–300 °C. A Kapton TPS sensor of radius 6.4 mm, a test specimen of size 50 mm square (on each side), and 25 mm in thickness are recommended for testing. The error in thermal conductivity measurements at higher temperatures using the ISO 22007-2 is around ±5% of the true value [32]. However, FRP composites, due to their nonhomogeneous nature, can have a larger variation in the measured properties. To verify the reliability of the measurements for the FRP specimen, thermal property tests should be repeated on at least three specimens from the same composition of the FRP.

#### 4.2.2. Specific Heat

Specific heat of conventional FRP composites can be measured using the differential scanning calorimetry (DSC) method specified in ASTM E1269 [28] and ISO 11357-4 [33] provisions. In this technique, the specific heat is measured based on the thermal energy needed to obtain a nearly zero temperature difference between the test specimen and a specimen of inert reference material. The DSC technique can be applied in the temperature range of 20 °C to 600 °C [28]. However, studies have shown that the error in specific heat measurements obtained using the DSC technique can be as high as ±20% [34].

ISO 22007-2 [32] recommends the hot disc method for the evaluation of specific heating, up to a temperature of 1000 °C. This hot disc method, described in Section 4.2.1 for thermal conductivity, can yield specific heat measurements. The same test equipment (the Hot Disk apparatus) and procedure as described above for thermal conductivity can be used for measuring specific heat as well. For the FRP specimens, the temperature range of interest for specific heat measurements is from 20 °C to 300 °C. Hot Disk TPS 2500S thermal constant analyzer is capable of simultaneously measuring the specific heat internally along with the thermal conductivity of the test specimen.

#### 4.2.3. Thermal Expansion

**Figure 2:**
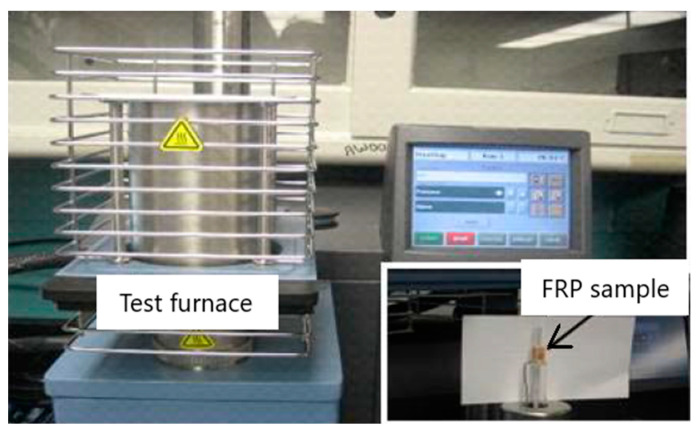
Experiment setup for thermo-mechanical analysis (TMA).

Standardized test methods for evaluating the thermal expansion of construction materials are specified in ASTM E831-19 [29] and ISO 11359-2 [35]. A thermo-mechanical analysis (TMA) technique can be utilized in these tests for room temperature and high-temperature measurements. In these tests, the coefficient of linear thermal expansion is determined by measuring the change in the length of the specimen with the temperature at a constant heating rate [30]. The two standard tests differ in technical aspects such as the maximum heating rate and specimen size. The test setup and equipment used for measuring the thermal expansion of the FRP composites are shown in Figure 2. Full details of the test equipment and procedure are reported in [30].

As per the provisions in ISO 11359-2 [35] and ASTM E831-19 [29] standards, the specimens to be used in the test should be between 5 to 10 mm in length with a strict constraint on the width not to exceed 10 mm. The FRP composites with a length of 10 mm and a transverse length (width or diameter) of 10 mm are recommended to capture the whole FRP composite including the fiber and matrix. Further, 3 °C per minute is to be selected as the temperature ramp for heating the FRP specimens [20]. The temperature range of the test is to be constrained to 20–300 °C, as FRP might decompose beyond 300 °C, which can damage the equipment. The apparatus continuously records the change in specimen length with temperature during the test. The coefficient of linear thermal expansion (α) between any two temperatures is calculated using the below formula [35]:

(1) $$\alpha =\frac{dL}{dT}\times \frac{1}{L_{0}}$$

where $L_{0}$ represents the sample length at room temperature, dL is the change in length at temperature T, and dT is the temperature change. To obtain reliable thermal expansion values, three repeat tests are to be conducted and the average of these measurements is to be taken as the coefficient of linear thermal expansion.

#### 4.2.4. Glass Transition Temperature

The test procedure for measuring the glass transition temperature (*T*_g_) of FRP composite materials is given in ASTM E1545-11 [36]. This method also uses a TMA technique to measure the change in the dimension of the specimen when the material is subjected to a constant heating rate through its glass transition phase. The operating temperature range for this technique is from 100 to 600 °C. This test method is similar to that in ISO 11359-2 [35]. For measuring the glass transition temperature, the specimen dimensions, equipment, and test procedure similar to that used for measuring the thermal expansion of the FRP specimen (as described in Section 4.2.3) can be utilized. However, a different sensing probe and heating rate are to be utilized.

Various sensing probes are available for use with the TMA technique, including expansion, penetration, compression, flexure, extension, and dilatometry, to achieve optimum test conditions for a specific specimen and/or application. For measuring the glass transition temperature, the softening stage of the FRP composite (from solid-state to viscous state) is to be captured and for this purpose, a penetration probe is to be used. A constant heating rate of 2 °C per minute is to be selected and the temperature range of the test is restricted between 20 to 300 °C [3]. The change in the length of the specimen versus temperature rise is recorded during the test and the temperature at which the slope of the displacement curve changes from expansion to penetration is taken as the glass transition temperature. Using a reduced heating rate increases the measured deflection at *T*_g_, reduces the signal-to-noise ratio and the transition is observed clearly [3]. The average *T*_g_ obtained from tests conducted on three samples is to be taken as the glass transition temperature of the FRP specimen. This procedure can be applied to conventional or bio-based FRP composites.

### 4.3. Mechanical Properties

The mechanical properties at high temperatures are highly sensitive to the testing regime (steady-state or transient state), specimen conditions (dimensions of the specimen, moisture content, etc.), loading parameters (heating rate and structural loading rate), and the instrumentation used for the tests. Test procedures for evaluating the mechanical properties of conventional FRP at ambient conditions are specified in the ASTM standards such as ASTM D7205/D7205M-06 [37] for tensile strength and modulus properties and ASTM D7913/D7913M-14 [38] for bond strength of FRP with concrete. However, limited studies have been carried out to characterize the mechanical properties of synthetic FRP at elevated temperatures using different test methods due to a lack of specific guidance in current standards [3,20]. These methods are adapted by extending the room temperature procedures to elevated temperature property evaluation. Based on a detailed review of these methods, recommendations are given below for measuring the temperature-dependent properties of bio-based FRP composites.

#### 4.3.1. Tensile Strength and Elastic Modulus

Due to the lack of specifications for standardized test methods in standards, a review of experimental methods adopted by researchers in prior studies for evaluating the tensile strength and stiffness properties of FRP was undertaken. High-temperature material property data are available to a limited extent on FRP composites used in aerospace and automobile industries. However, such data are more scarce in the case of FRP composites used in construction applications [5]. Moreover, the mechanical property tests were carried out in the temperature range of 20–300 °C [39,40,41], which is not sufficient to evaluate the overall performance of the FRP under the full temperature range, as encountered in a fire. Very few tests focused on the strength and stiffness degradation of specific FRP bars, strips, plates, and other coupons in the temperature range of 20 to 600 °C.

Based on the available published information, the test method adopted by Yu and Kodur [4] to evaluate the strength and stiffness properties of the FRP rebars and strips at room and elevated temperatures up to 600 °C seems to be reliable and practical. This study also indicates that undertaking strength tests on FRP specimens at elevated temperatures is rather complicated and tricky due to numerous issues including gripping (anchorage), burning, and softening of FRP materials. Based on numerous trials, the researchers have recommended a few guidelines for undertaking strength and bond tests.

**Figure 3:**
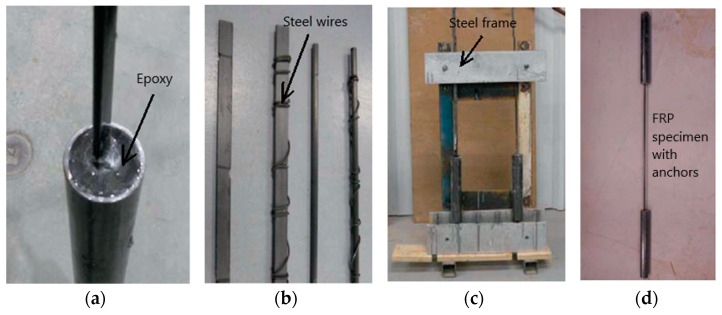
Fabrication of anchor system for FRP specimens indicating (**a**) epoxy filling, (**b**) steel wires, (**c**) steel frame, and (**d**) test specimen.

In a tension test, the FRP specimens are susceptible to crushing under the pressure applied due to gripping. Strong anchors are to be provided at the two ends of the FRP test specimen to ensure that the failure of the specimen occurs in the central region experiencing high temperature, rather than at cooler ends (or anchorage zone). Anchor systems in FRP coupons play a critical role in the test results (and also the success of the test even) and for this purpose anchor system is to be provided as per the ACI 440.3 [42] specifications for room temperature testing and those recommended by Wang et al. for high-temperature testing [19]. Proper anchorage can be achieved through filling high-strength adhesive into a circular steel tube (confinement), as shown in Figure 3. The filling material that can be used for the anchors includes high-strength epoxy and expansive cement, although the expansive cement was found to provide a better bond and negligible slip during the experiment [4]. Further details on the preparation of epoxy and filling material can be found in [4].

To confine the filling materials, steel pipes with dimensions per ACI 440.3 [42] specifications are to be used. To prevent sliding of the FRP specimen, small dents are to be created on the bars and strips, and steel wires are to be bound to these dents. Care must be taken to ensure that the FRP specimen is aligned perfectly vertical while the epoxy (or cement) is filled into the tube. This is to avoid any eccentric forces that can be generated during the tension test. For this purpose, a steel or wooden frame (as shown in Figure 3) can be fabricated to align the FRP specimen and tube in the vertical direction. The procedure to mount the FRP specimens in the frame is discussed in [4]. Alraie et al. [43] have also given a description of the development of anchors for basalt fiber-reinforced polymer rods.

**Figure 4:**
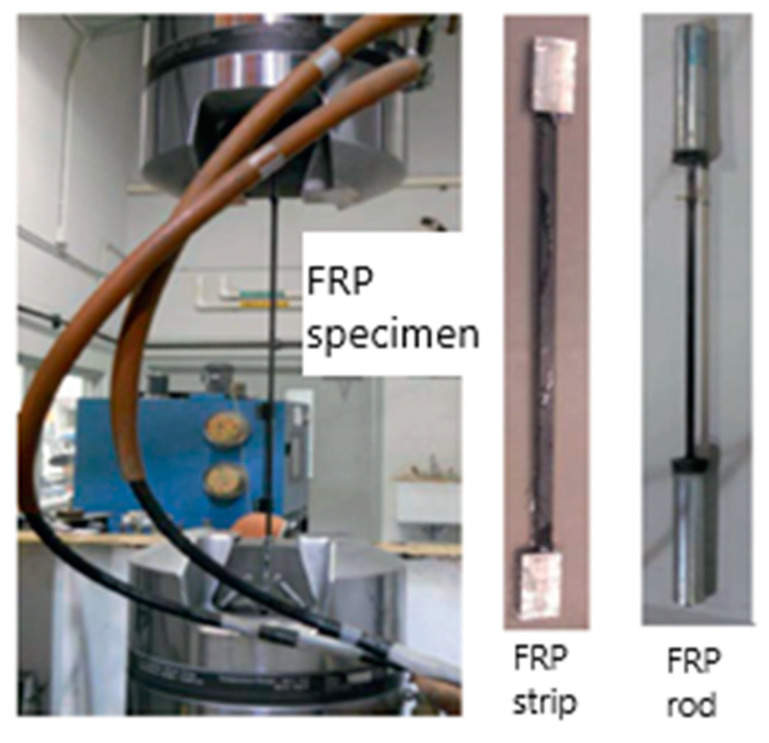
Test apparatus and FRP composite specimen placement for room temperature strength test.

**Figure 5:**
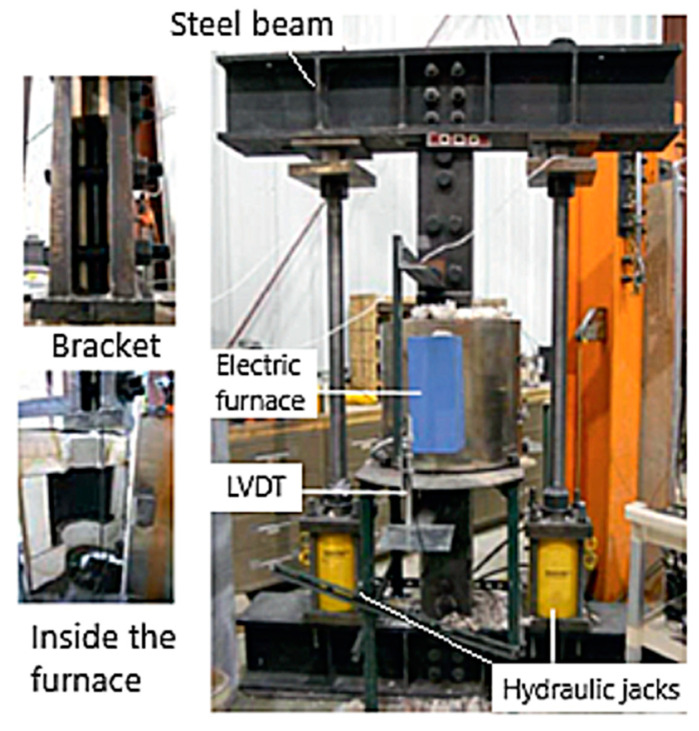
Test setup for FRP composite strength test at elevated temperatures.

For the room temperature strength tests, the hydraulic Materials Test System (MTS) is used (as shown in Figure 4). The MTS machine is suitable for the application of high tensile loads and provides the necessary grip to FRP specimens for the measurement of strength and stiffness at room temperature [4]. For the high-temperature tests, a test setup similar to the one shown in Figure 5 is recommended. The test setup comprises the FRP specimen (with the anchor system), an electric furnace, two pairs of clamping brackets to hold the specimen, and two steel beams at the top and bottom. The test is carried out by first heating the FRP specimen to a target temperature. The temperature in the specimen is recorded using thermocouples installed at mid-height and quarter-height of the specimen. The heating rate to be used for the test can be set to 5 to 10 °C/min, depending on the target temperature, with the faster rate chosen for reaching higher target temperatures.

Once the target temperature is attained in the specimen, the hydraulic jacks are used to apply the tensile load by varying the distance between the top and bottom beams. The elongation of the specimen is measured using a linear variable differential transformer (LVDT), which is to be attached between the upper and lower clamping brackets. The loading is incremented until the specimen elongates considerably and fails. Data from the tension tests carried out on the FRP specimens can be used to evaluate the tensile strength, elastic modulus, and stress–strain relations at various temperatures. The tensile strength at a tested temperature is taken as the maximum load at failure divided by the actual cross-sectional area of the test specimen, whereas the slope of the linear portion of the stress–strain curve is taken as the elastic modulus. At each target temperature, at least two tension tests are to be conducted, and the average of the two (or more) values is taken as tensile strength and elastic modulus of the tested FRP specimen.

#### 4.3.2. Bond Strength

The information on bond strength and bond-slip relations are critical for evaluating the capacity and deformations in an FRP component or FRP-reinforced concrete member. The bond strength of FRP can be measured using pull-out tests conducted at room temperature conditions. Although there are standardized test procedures for evaluating bond strength at ambient temperatures, no guidance is available in the current standards for high-temperature tests. Similar to strength tests, bond tests at elevated temperatures are quite complex due to issues such as gripping and softening, and burning of the FRP composite, specifically the matrix. In the literature, very few tests are reported at elevated temperatures, especially in the temperature range experienced by the FRP composite member during fire conditions. Of these methods, the test method by Yu and Kodur [2] to evaluate the bond strength of near-surface mounted (NSM) FRP specimens in the temperature range of 20 to 400 °C is practical and seems to yield reasonable data. This study specifies the much-needed guidelines for specimen preparation, design of grips (or anchor system), and testing regime needed for bond strength measurement at high temperatures.

**Figure 6:**
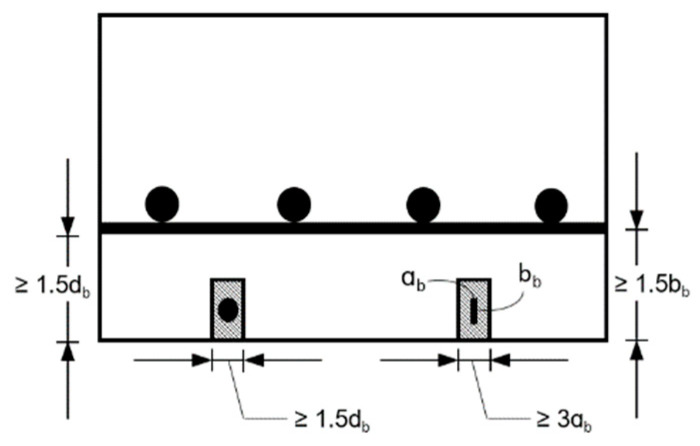
Groove size for installation of NSM FRP.

For preparing the specimens for bond strength test, concrete blocks of 150 × 150 × 400 mm are to be cast from a batch of premixed concrete. FRP specimens in the form of bars or strips can be tested using this method. One end of the specimen is to be bonded with the concrete block while the other end is to be installed with a strong anchor system that facilities gripping of the FRP specimen during the pull-out tests. The anchor system is to be designed as per the ACI 440.3 [42] specifications at room temperature and specifications by Wang et al. [19] at elevated temperatures, similar to that used in the tension test, and the details of the anchor are given in Section 4.3.1. To install the FRP composite coupon in the concrete block, a groove is to be cut out from the concrete block, as shown in Figure 6. The groove size is taken based on the recommendations made in the ACI 440.3 [42]. A bond length of 150 mm is specified for all FRP specimens. Recommendations specified by Yu and Kodur [2] are to be followed to attach the FRP to the concrete block.

**Figure 7:**
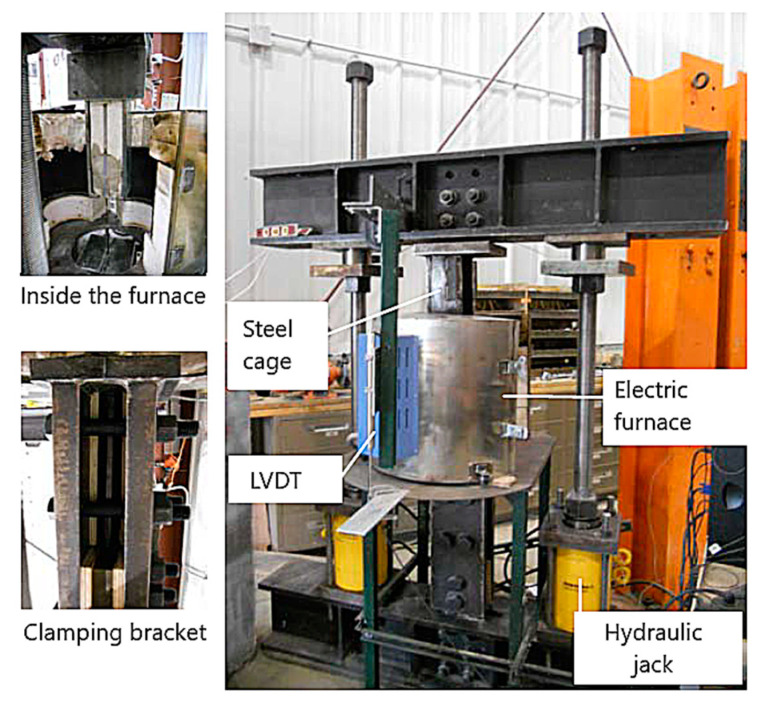
Test setup for evaluating bond strength of NSM FRP systems at high temperatures.

The test equipment for carrying out the high-temperature bond tests is comprised of a tension-testing machine and an electric furnace to subject the specimen to elevated temperatures, as shown in Figure 7. This setup is similar to the tension-testing equipment described in Section 4.3.1. For the pull-out tests, the end of the specimen which is embedded in the concrete block is held by a steel cage which is then connected to the top beam. The other end with the anchor system is clipped to a pair of clamping brackets, which are then connected to the bottom beam. Through this setup, the pull-out test can be carried out by subjecting the FRP specimen to heat to a specified temperature followed by tensile loading. The procedure and heating rates specified for the tensile strength test (in Section 4.3.1) are to be followed for heating the FRP specimen to a target temperature. To measure the temperature inside and outside of the epoxy, two thermocouples, with one placed inside the groove and the other on the concrete surface, are recommended. 

Once the specimen attains the target temperature, the pull-out test is to be carried out by applying a tensile load using hydraulic jacks. The velocity of the loading is to be set at 2 mm/min. The slip between the FRP reinforcement and concrete block is measured using an LVDT that is to be placed between the top beam and the clamping brackets. The displacement of the top beam is to be continued until the FRP strip, rod, or plate is pulled out. The peak average bond stress (bond strength, $\tau_{max}$) and bond modulus (E) are calculated as:

(2) $$\tau_{max}=P_{max}/A$$

(3) $$E=\Delta \tau /\Delta \varepsilon_{slip}$$

where $P_{max}$ is the maximum load recorded during the pull-out test, A is the contact area between the FRP and epoxy, Δτ and $\Delta \varepsilon_{slip}$ are the relative bond stress and slip strain on the linear part of the bond stress–strain curve [2].

The slip strain is calculated as:

(4) $$\varepsilon_{slip}=s/L_{bond}$$

where s is the slip measured during the test, and $L_{bond}$ is the bond length of the test specimen. At each target temperature, two pull-out tests are to be carried out and the average of the two values is taken as the bond strength and modulus of the FRP system.

## 5. Case Study: Application of Test Methods for Characterizing High-Temperature Properties of FRP Composites

To illustrate the applicability of the test procedures recommended above in evaluating the properties of the FRP composites, some of the elevated temperature property tests carried out at Michigan State University (MSU) on the FRP composites are presented. These material property tests were carried out at various target temperatures on specimens made of the conventional FRP composites and following the recommended test methods discussed in the previous sections. Only the main results are presented in this section and detailed information on the test program and results are reported in other reports and thesis [2,3,4,20].

### 5.1. Thermal Property Measurements

#### 5.1.1. Thermal Expansion

To illustrate the thermal expansion tests on FRP composites at elevated temperatures, tests carried out on four types of commercially available FRP reinforcement that are used in NSM strengthening applications are presented here. Table 4 summarizes the dimensions and manufacturer-specified properties of the FRP samples used. The fibers in these composites were glass and carbon fibers and the matrix was vinyl ester resin in Aslan glass fiber-reinforced concrete (GFRP) and Aslan carbon fiber-reinforced polymer (CFRP) specimens while Tyfo S and Tyfo 300 epoxy matrix were used in the Tyfo CFRP strips and rods. For the test, the samples were trimmed such that the specimen dimensions were restricted to 10 mm in length and 10 mm in the transverse direction (width or diameter). The thermal expansion tests were carried out using the TMA equipment as per the test procedure recommended in Section 4.2.3. Two repeat tests were carried out for each specimen and the average of the readings is taken as the coefficient of thermal expansion of the FRP specimen.

**Figure 8:**
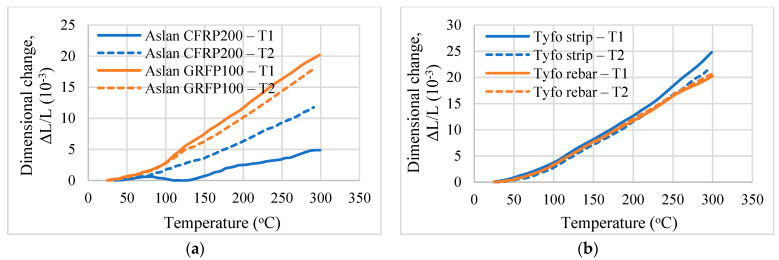
Thermal expansion of NSM FRP specimens in the transverse direction. (**a**) Thermal expansion of Aslan GFRP and Aslan CFRP. (**b**) Thermal expansion of Tyfo rods and Tyfo strips.

**Figure 9:**
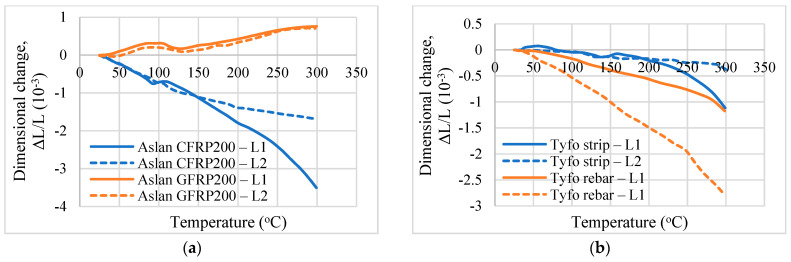
Thermal expansion of NSM FRP specimens in the longitudinal direction. (**a**) Thermal expansion of Aslan GFRP and Aslan CFRP. (**b**) Thermal expansion of Tyfo rods and Tyfo strips.

Measured dimensional changes in the transverse and longitudinal directions of the FRP specimens are shown in Figure 8 and Figure 9. T1 and T2 indicate the dimensional changes in the transverse direction, whereas L1 and L2 indicate the dimensional changes in the longitudinal direction measured from the two repeat tests, respectively. It is seen that the recommended test method can capture the variation of change in dimension in the temperature range of 20 to 300 °C. A larger variation in length is observed in the transverse direction since the properties of the polymer matrix control the thermal expansion in this direction, and the polymer matrix undergoes higher expansion with temperature rise. The test results also indicate that the thermal expansion of FRP with changing fibers (glass and carbon) is different. The dimensional changes in the longitudinal direction for the CFRP specimens are mostly negative which indicates that the CFRP shrinks with temperature rise. This is in context to the GFRP specimens, which undergo expansion at elevated temperatures, as seen in Figure 9. Detailed discussions on the different thermal expansion trends measured from the test program are reported in [20].

From the measured dimensional changes, the coefficient of thermal expansion at different temperatures is computed using Equation (1) and is tabulated in Table 5. It is seen that the coefficient of thermal expansion varies significantly with the composition of the NSM FRP and direction. These measured thermal expansion results are in the same trend as that of the other conventional FRP composites used in aerospace applications. Based on the reported test data, it is recommended to evaluate the coefficient of thermal expansion of bio-based FRP for a wide temperature range of 20 to 300 °C using different fibers in both transverse and longitudinal directions.

#### 5.1.2. Glass Transition Temperature

For illustrating glass transition temperature (*T*_g_) tests on FRP composites used in construction applications, high-temperature tests were carried out on Tyfo SCH-41 FRP specimen which comprised of unidirectional carbon fabric with glass cross fibers in an epoxy matrix [3]. The ultimate tensile strength in the primary fiber direction, elongation at break, and tensile modulus of the composite laminate were 986 MPa, 1%, and 95.8 Gpa, respectively. The tensile strength and tensile modulus of the epoxy were 72.4 Mpa and 3.18 Gpa, respectively, while the flexural strength and flexural modulus of the epoxy were 123.4 Mpa and 3.12 Gpa, respectively. The supplier of the FRP had reported a *T*_g_ value of 82 °C based on the ASTM D-4065 standard test dynamic mechanical analysis (DMA) [44]. In this test program, the procedure specified in Section 4.2.4 is used and the TMA equipment was utilized for these tests. The test procedure was repeated for two different heating rates of 2 °C/min and 10 °C/min to observe the effect on the test results.

**Figure 10:**
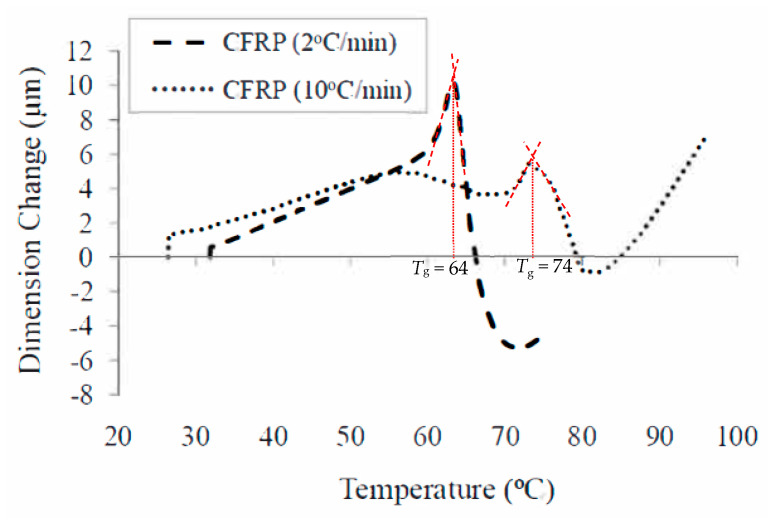
Variation of *T*_g_ in CFRP composites as a function of temperature and heating rate.

Figure 10 shows the measured dimensional change in the specimen as a function of temperature for the two heating rates used in the test program. The reduced heating rate increased the measured deflection at *T*_g_, thus decreasing the signal-to-noise ratio and the transition temperature was shifted downward. This is illustrated in Figure 10 where a faster heating rate (10 °C/min) gives a higher value of *T*_g_ and the average penetration of the probe drops [3]. Based on this, a lower heating rate of 2 °C/min is recommended for measuring the glass transition temperature of typical FRP composites used in construction applications. Moreover, the measured *T*_g_ of 64 °C is about 25% lower than the *T*_g_ reported by the manufacturer. This is mainly because the glass transition temperature provided by the manufacturer was determined by using a DMA, which depends not only on the heating rate but also depends on the frequency used in the test. Further, the varying atmospheric conditions and moisture content affect the glass transition temperature [3], and these effects are not reversible. Since the test parameters such as frequency and moisture content were not reported by the manufacturer, it is not possible to replicate the original *T*_g_ value reported by the manufacturer.

### 5.2. Mechanical Property Measurements

#### 5.2.1. Tensile Strength Tests

For undertaking tests to evaluate the tensile strength, elastic modulus, and stress–strain relations of FRP at elevated temperatures, 25 CFRP specimens were utilized [4]. In total, 13 of these test specimens were CFRP strips of size 4.5 mm × 13.5 mm, while the remaining 12 were CFRP rods of 6.4 mm diameter. In both cases, the composite was composed of carbon fibers embedded in an epoxy resin. The carbon fiber content in the strips and rods was 62% and 60%, respectively. The nominal tensile strength and modulus of the CFRP strip at room temperature, as specified by the manufacturer, were 2790 MPa and 155 GPa, respectively, and the ultimate tensile strain is 0.018. For the CFRP rod, the corresponding nominal tensile strength, elastic modulus, and ultimate strain were 2070 MPa, 124 GPa, and 0.017, respectively. Other properties of the FRP specimens used in the test program can be found in Yu and Kodur [4]. Tensile strength tests at room temperature and elevated temperatures were carried out using the test procedure recommended in Section 4.3.1.

From the room temperature tests, the tensile strength and elastic modulus of NSM CFRP strips were found to be 1641 MPa and 150.8 GPa, while the corresponding values for the CFRP rods were found to be 1577 MPa and 130.9 GPa, respectively. While the elastic modulus of the CFRP specimens measured in the tests was comparable to those specified by the manufacturer, the room temperature tensile strengths were much lower than the strengths specified by the manufacturer. This is mainly because the manufacturer used only the strength of the carbon fibers in determining the tensile strength; however, in the tension test, CFRP (composite) specimens did not attain this strength due to the degradation (and fracture) of the epoxy resin [4]. Thus, the tensile strength obtained from the room temperature test provides a more accurate strength measurement of FRP composites, as the test specimen comprises both fibers and resin, as used in construction applications.

**Figure 11:**
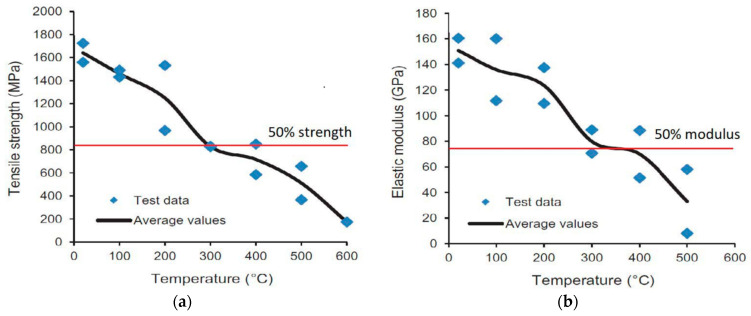
Variation of tensile strength and elastic modulus of CRFP strips with temperature. (**a**) Tensile strength; (**b**) Elastic modulus.

**Figure 12:**
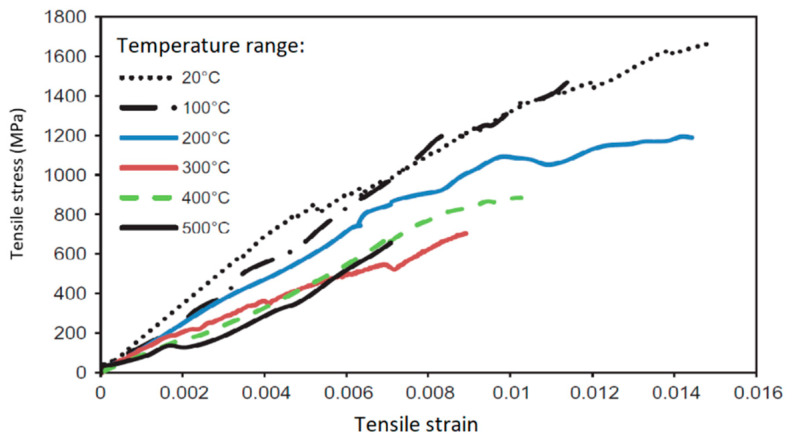
Stress–strain response of CFRP strips at different temperatures.

**Figure 13:**
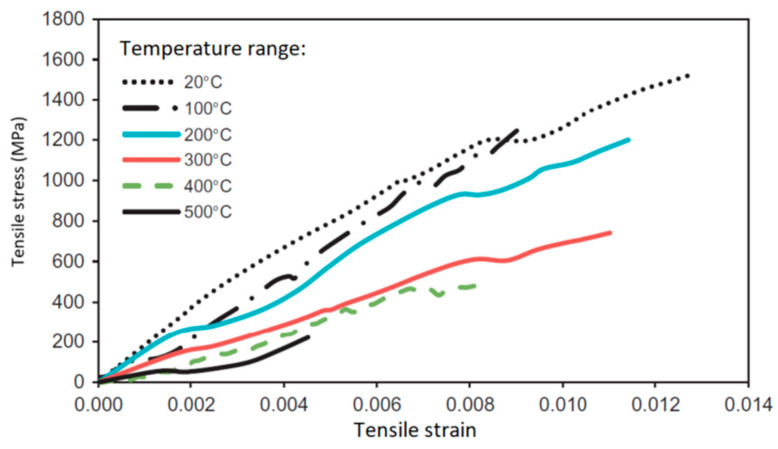
Stress–strain response of CFRP rods at different temperatures.

The reduction in elastic modulus and tensile strength of the CFRP strips with temperature is shown in Figure 11. The degradation of modulus and strength of the CFRP specimens are gradual between 20 °C and 300 °C, and then rapid degradation occurs beyond 300 °C. This is attributed to the decomposition of the polymer resin at around 300 °C. The tensile strength of the CFRP specimens drops to 50% around 300 °C and this temperature can be considered the critical temperature for the CFRP specimens. From the test measurements, the stress–strain relations are plotted for various CFRP specimens at different elevated temperatures as shown in Figure 12 and Figure 13. As in the case of other materials, the stress–strain response of the tested FRP degrades with temperature, with lower strength at increasing temperature. However, the ultimate strain does not follow the increasing trend with temperature. This is unlike the stress–strain response of other construction materials. This test method can also be applied to bio-based FRP composites to establish the critical temperature and stress–strain relations for bio-based FRP materials at elevated temperatures.

#### 5.2.2. Bond Strength Tests

**Figure 14:**
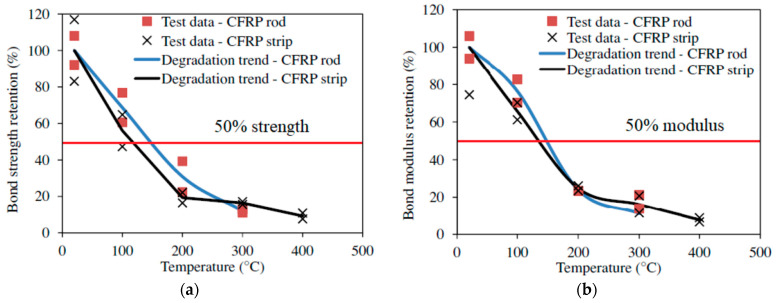
Variation of bond strength and elastic modulus of NSM CFRP strips and rods with Tyfo T300 epoxy with temperature; (**a**) Bond strength; (**b**) Bond modulus.

**Figure 15:**
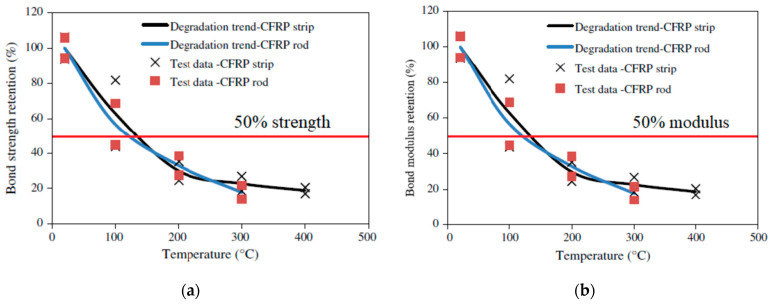
Variation of bond strength and elastic modulus of NSM CFRP strip and rod with Tyfo S epoxy with temperature; (**a**) Bond strength; (**b**) Bond modulus.

To evaluate the bond properties of FRP composites, 36 pull-out tests were carried out on NSM FRP-strengthened concrete specimens at various temperatures [2]. The specimens consisted of CFRP strips and rods embedded in two types of adhesives, namely Tyfo S epoxy and T300 epoxy. The properties of the specimens used in the test program are tabulated in Table 6. The concrete blocks used in the test were 150 × 150 × 400 mm in size and a batch of pre-mixed concrete comprising Type I Portland cement, sand, and carbonate-based coarse aggregate was used to cast the blocks. The measured 28-day and 90-day compressive strengths of concrete were 48 MPa and 50 MPa, respectively. The anchor system and the groove on the concrete block were designed as discussed in Section 4.3.2. Further details regarding the specimen preparation can be found in Yu and Kodur [2]. The specialized test setup and the procedure recommended in Section 4.3.2 were utilized to carry out the bond strength tests at elevated temperatures. The normalized bond strength and bond modulus at different temperatures evaluated from the pull-out tests on the NSM FRP specimens are shown in Figure 14 and Figure 15.

In all the NSM FRP specimens, the bond strength and modulus degraded rapidly with an increase in temperature. The degradation of bond strength occurs at a faster pace in the 20 to 200 °C temperature range and at 200 °C, the NSM FRP retained only 20 to 30% of its original strength. The rapid degradation is attributed to the softening of the epoxy matrix beyond the glass transition temperature (around 80 °C) [2]. Above 200 °C the rate of deterioration is low since the FRP has lost most of its strength by this stage. Observations from the tests indicate that at around 400 °C, the epoxy starts to burn, which damages the bond between FRP and concrete. The bond strength becomes negligible at 400 °C for the CFRP strips and at 300 °C for the CFRP rods and the tests were terminated at this temperature. As explained above, several complexities (including gripping issues, flaming of specimens, etc.) were encountered in this test program and the solutions to overcome these challenges are discussed in detail by Yu and Kodur [2].

**Figure 16:**
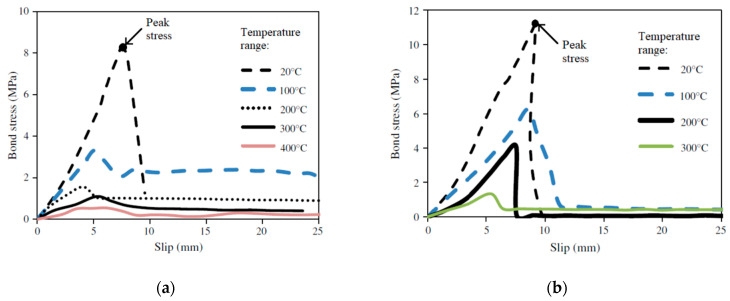
Bond stress–slip relations for NSM CFRP specimens with Tyfo 300 epoxy at different temperatures; (**a**) NSM CRFP strip. (**b**) NSM CFRP rod.

**Figure 17:**
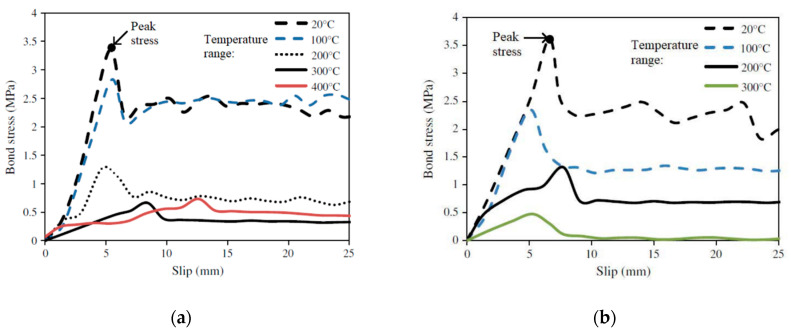
Bond stress–slip relations for NSM CFRP specimens with Tyfo S epoxy at different temperatures; (**a**) NSM CFRP strip; (**b**) NSM CFRP rod.

The bond stress–slip relations at different temperatures for the different NSM FRP specimens are shown in Figure 16 and Figure 17. Irrespective of the type of epoxy resin and the shape of the reinforcement, a similar stress–slip response is obtained in all the pull-out tests. The bond stress–slip response can be critical in evaluating the actual slip and hence, the force transfer between the FRP and concrete in an FRP-strengthened structural member exposed to elevated temperatures, which is effectively captured in the test program. Thus, the test method recommended in this paper can also be applied to bio-based FRP to evaluate the degradation of bond strength and modulus and the bond stress–slip relations at elevated temperatures.

As seen from the presented case studies; the recommended testing protocols are able to capture the variation of the thermal and mechanical properties of the FRP composite at elevated temperatures. Additionally, the recommended procedures for specimen preparation, fabrication of anchors (grips), and heating (and loading) regimes are practical and efficient for measuring the different properties of the FRP composite at elevated temperatures

## 6. Applicability of Test Methods to Bio-Based FRP Composites

The recent focus in construction is to adopt bio-based FRP composites since it has numerous benefits including sustainability, environmental friendliness, low cost, locally sourced raw materials, etc. Temperature-dependent properties of FRP composites are critical for evaluating the fire resistance of standalone FRP components, and FRP-reinforced and FRP-strengthened concrete members. Preliminary studies clearly show that there is a larger variability in the properties of the FRP components made using natural fibers (bio-based) even at room temperature. At elevated temperatures, these plant-based FRP composites are susceptible to flaming, charring, and rapid loss of strength and bond properties, much like the conventional FRP composites. Moreover, the specimen conditions, such as the age of the specimen and moisture content, testing regime, and rates of loading and heating can vary significantly due to varying types of plants used, such as fiber, and this can have a high influence on the properties at ambient and elevated temperature conditions. For undertaking property evaluation, standardized test methods are critical for evaluating the properties of bio-based FRP composites.

In the present study, a review of the various test methods adopted by researchers for the evaluation of thermal and mechanical properties of synthetic FRP composites is conducted. Upon detailed review, the most suitable test procedures for characterizing critical material properties of the FRP at high temperatures are recommended. The case study on conventional FRPs shows that the recommended test procedures can capture the variation in thermal and mechanical properties at elevated temperatures. Since the bio-based composites are of similar construction (comprising of fibers and resin) as that of the synthetic composites, the procedures and the recommended guidelines for specimen conditions, loading and heating rates, anchor systems, etc. can also be applied to undertake property tests on bio-based FRP composites for generating reliable material property data in the range of 20 °C to 600 °C. High-temperature property data compiled from these tests can be utilized to input relevant property relations in numerical models for predicting the performance of bio-based FRP under different fire exposure conditions.

## 7. Conclusions

A review of the test methods and procedures for evaluating thermal and mechanical properties of FRP composites at elevated temperatures is conducted and presented. Based on the reviewed literature, recommendations on the most suitable test methods for the high-temperature material characterization of the FRPs have been made.

1. There is a lack of standardized test methods and procedures in current codes and standards for the characterization of high-temperature properties of synthetic and bio-based FRP in the temperature range of 20 °C to 600 °C.
2. There are limited reported data on high-temperature properties and associated material property relations for conventional FRP composites and no such data are available for newer FRP composites incorporating bio-based FRP composite material.
3. The recommended test procedures based on the review of the test methods in current standards and published literature can be adopted for the characterization of material properties of conventional and bio-based FRP composites in the temperature range of 20 °C to 600 °C.
4. The case study shows that the recommended test procedures for the characterization of thermal and mechanical properties of FRP composites can be used to evaluate the high-temperature properties of the FRP composites reliably.

## Figures and Tables

**Table 1:** Room temperature material properties of synthetic and natural fibers and their composites.

Fiber or Composite	Density (g/cm^3^)	Tensile Strength (MPa)	Elastic Modulus (GPa)	Elongation at Break (%)
Carbon	1.7	4000	230–240	1.4–1.8
E-glass	2.5	2000–3500	70	2.5
S-glass	2.5	4570	86	2.8
Aramid	1.4	3000–3150	63–67	3.3–3.7
Cotton	1.5–1.6	287–800	5.5–12.6	7.0–8.0
Jute	1.3–1.45	393–773	13–26.5	1.16–1.5
Flax	1.5	345–1100	27.6	2.7–3.2
Hemp	1.48	550–900	70	1.6
Sisal	1.45	468–640	9.4–22	3–7
Carbon/epoxy	1.5–2.1	1050–1500	180	0.5–1.8
Glass/epoxy	1.25–2.5	700–1050	42–55	1.2–5
Aramid/epoxy	1.25–1.45	1400	76	1.4–4.4
Jute/unsaturated polyester	-	50	8	-
Flax/epoxy	-	132–160	15–27	-
Hemp/polypropylene	-	52	4	
Sisal/epoxy	-	330–410	6–10	-
Kenaf/polypropylene	-	46	5	-

**Table 2:** Test standards and temperature range for the evaluation of thermal properties of FRP.

Thermal Property	Temperature Range	Test Standards
Thermal conductivity	Ambient temperature to 8 °C	ASTM C177, ASTM C1363, and ISO 8302
	Elevated temperature (20 °C to 300 °C)	ISO 22007-2
Specific heat	Ambient temperature to 300 °C	ASTM E1269, ISO 11357-4, and ISO 22007-2
Thermal expansion	Ambient temperature to 300 °C	ASTM E831-19 and ISO 11359-2
Glass transition temperature	Ambient temperature to 300 °C	ASTM E1545-11 and ISO 11359-2

**Table 3:** Test standards and temperature range for the evaluation of mechanical properties of FRP.

Mechanical Property	Temperature Range	Test Standards
Tensile strength and elastic modulus	Ambient temperature	ASTM D7205/D7205M-06
	Elevated temperature (20 °C to 600 °C)	Yu and Kodur [4]
Bond strength	Ambient temperature	ASTM D7913/D7913M-14
	Elevated temperature (20 °C to 400 °C)	Yu and Kodur [2]

**Table 4:** Properties of NSM FRP specimens used for the thermal expansion test.

FRP Specimens	Dimensions (mm)		Fiber Content (%)	*T_g_* (°C)
	Section	Length		
Aslan GFRP 100	dia. 9	10/10	>70 (weight)	>110
Aslan CFRP 200	dia. 13	10/10	N/A	>110
Tyfo CFRP strip	13.5 × 4.5	10/10	62 (volumetric)	71
Tyfo CFRP rebar	dia. 6	10/10	60 (volumetric)	71

**Table 5:** Longitudinal and transverse coefficients of thermal expansion of NSM FRP reinforcement.

NSM FRP Specimen		Longitudinal Direction (10^−6^/°C)			Transverse Direction (10^−6^/°C)		
		Δ*T* = 50 °C	Δ*T* = 100 °C	Δ*T* = 280 °C	Δ*T* = 50 °C	Δ*T* = 100 °C	Δ*T* = 280 °C
Aslan GFRP 100	Test 1	3.5	1.3	2.6	28.2	52.9	74.8
	Test 2	5.3	1.7	2.8	28.0	49.9	68.5
	Average	4.4	1.5	2.7	28.1	51.4	71.7
Aslan CFRP 200	Test 1	−10.3	−8.4	−13.0	10.6	12.1	18.3
	Test 2	−9.5	−9.7	−6.3	16.4	28.8	44.1
	Average	−9.9	−9.1	−9.6	13.5	20.5	31.2
Tyfo CFRP rod	Test 1	−1.7	−2.9	−4.3	37.6	56.9	67.4
	Test 2	−5.9	−7.5	−10.3	35.9	60.0	68.3
	Average	−3.8	−5.2	−7.3	36.7	58.5	67.8
Tyfo CFRP strip	Test 1	0.6	−0.8	−3.7	40.5	59.4	82.9
	Test 2	−0.0.4	−1.02	−1.2	26.4	51.1	73.0
	Average	0.1	−1.0	−2.5	33.4	55.2	77.9

**Table 6:** Properties of NSM FRP specimens used in the bond test program.

Test Groups	NSM Adhesive	Reinforcement				Tested Temperature Range (°C)
		Type	Size (mm)	Strength (MPa)	Modulus (GPa)	
I	Tyfo T300	CFRP strip	4 × 13.5	2790	155	20–400
II	Tyfo T300	CFRP rod	Dia. 6	2070	124	20–300
III	Tyfo S	CFRP strip	4 × 13.5	2790	155	20–400
IV	Tyfo S	CFRP rod	Dia. 6	2070	124	20–300

## Data Availability

Some or all data, models, or codes that support the findings of this study are available from the corresponding author upon reasonable request. Specifications of testing equipment and measured data from experiments are used in figures and tables.

## References

[1] Kodur V., Naser M. (2020). Structural Fire Engineering.

[2] Yu B., Kodur V. (2014). Effect of high temperature on bond strength of near-surface mounted FRP reinforcement. Compos. Struct..

[3] Ahmed A. (2010). Behavior of FRP-Strengthened Reinforced Concrete Beams under Fire Conditions.

[4] Yu B., Kodur V. (2014). Effect of temperature on strength and stiffness properties of near-surface mounted FRP reinforcement. Compos. Part B Eng..

[5] Kodur V., Bhatt P., Naser M. (2019). High temperature properties of fiber reinforced polymers and fire insulation for fire resistance modeling of strengthened concrete structures. Compos. Part B Eng..

[6] Mohanty A., Misra M., Hinrichsen G. (2000). Biofibres, biodegradable polymers and biocomposites: An overview. Macromol. Mater. Eng..

[7] Pandey J., Nagarajan V., Mohanty A., Misra M. (2015). Commercial potential and competitiveness of natural fiber composites. Biocomposites.

[8] Lau K., Hung P., Zhu M.-H., Hui D. (2018). Properties of natural fibre composites for structural engineering applications. Compos. Part B Eng..

[9] Akil H.M., Santulli C., Sarasini F., Tirillò J., Valente T. (2014). Environmental effects on the mechanical behaviour of pultruded jute/glass fibre-reinforced polyester hybrid composites. Compos. Sci. Technol..

[10] Mochane M.J., Mokhena T.C., Mokhothu T.H., Mtibe A., Sadiku E.R., Ray S.S., Ibrahim I.D., Daramola O.O. (2019). Recent progress on natural fiber hybrid composites for advanced applications: A review. Express Polym. Lett..

[11] Chaudhary V., Bajpai P.K., Maheshwari S. (2018). Studies on mechanical and morphological characterization of developed jute/hemp/flax reinforced hybrid composites for structural applications. J. Nat. Fibers.

[12] Fan M., Fu F. (2017). Introduction: A perspective–natural fibre composites in construction. Advanced High Strength Natural Fibre Composites in Construction.

[13] Ticoalu A., Aravinthan T., Cardona F. A review of current development in natural fiber composites for structural and infrastructure applications. Proceedings of the Southern Region Engineering Conference (SREC 2010), Engineers Australia.

[14] Steffens F., Steffens H., Oliveira F.R. (2017). Applications of natural fibers on architecture. Procedia Eng..

[15] Padanattil A., Karingamanna J., Mini K.M. (2017). Novel hybrid composites based on glass and sisal fiber for retrofitting of reinforced concrete structures. Constr. Build. Mater..

[16] Berardi U., Iannace G. (2015). Acoustic characterization of natural fibers for sound absorption applications. Build. Environ..

[17] Ahmed A., Guo S., Zhang Z., Shi C., Zhu D. (2020). A review on durability of fiber reinforced polymer (FRP) bars reinforced seawater sea sand concrete. Constr. Build. Mater..

[18] Pickering K.L., Efendy M.A., Le T.M. (2016). A review of recent developments in natural fibre composites and their mechanical performance. Compos. Part A Appl. Sci. Manuf..

[19] Wang Y.C., Wong P., Kodur V. (2007). An experimental study of the mechanical properties of fibre reinforced polymer (FRP) and steel reinforcing bars at elevated temperatures. Compos. Struct..

[20] Yu B. (2013). Fire Response of Reinforced Concrete Beams Strengthened with Near-Surface Mounted FRP Reinforcement.

[21] Kodur V., Harmathy T. (2016). Properties of building materials. SFPE Handbook of Fire Protection Engineerin.

[22] (2021). Standard Test Method for Surface Burning Characteristics of Building Materials.

[23] (2006). Standard Method of Test of Surface Burning Characteristics of Building Materials.

[24] (2019). Standard Test Method for Specific Optical Density of Smoke Generated by Solid Materials.

[25] (2016). Standard Test Method for Surface Flammability of Materials Using a Radiant Heat Energy Source.

[26] (2019). Standard Test Method for Steady-State Heat Flux Measurements and Thermal Transmission Properties by Means of the Guarded-Hot-Plate Apparatus.

[27] (2019). Standard Test Method for Thermal Performance of Building Materials and Envelope Assemblies by Means of a Hot Box Apparatus.

[28] (2018). Standard Test Method for Determining Specific Heat Capacity by Differential Scanning Calorimetry.

[29] (2019). Standard Test Method for Linear Thermal Expansion of Solid Materials by Thermomechanical Analysis.

[30] Kodur V.K., Banerji S., Solhmirzaei R. (2020). Test methods for characterizing concrete properties at elevated temperature. Fire Mater..

[31] (1991). Thermal Insulation—Determination of Steady-State Thermal Resistance and Related Properties—Guarded Hot Plate Apparatus.

[32] (2015). Plastics—Determination of Thermal Conductivity and Thermal Diffusivity—Part 2: Transient Plane Heat Source (Hot Disc) Method.

[33] (2021). Plastics—Differential Scanning Calorimetry (DSC)—Part 4: Determination of Specific Heat Capacity.

[34] (2018). Structural Fire Engineering.

[35] (1999). Plastics—Thermomechanical Analysis (TMA)—Part 2: Determination of Coefficient of Linear Thermal Expansion and Glass Transition Temperature.

[36] (2016). Standard Test Method for Assignment of the Glass Transition Temperature by Thermomechanical Analysis.

[37] (2016). Standard Test Method for Tensile Properties of Fiber Reinforced Polymer Matrix Composite Bars.

[38] (2020). Standard Test Method for Bond Strength of Fiber-Reinforced Polymer Matrix Composite Bars to Concrete by Pullout Testing.

[39] Dimitrienko Y.I. (1998). Thermomechanics of Composites under High Temperatures.

[40] Fujisaki T., Nakatsuji T., Sugita M. (1993). Research and development of grid shaped FRP reinforcement. Spec. Publ..

[41] Kumahara S., Masuda Y., Tanano H., Shimizu A. (1993). Tensile strength of continuous fiber bar under high temperature. Spec. Publ..

[42] (2012). Guide Test Methods for Fiber-Reinforced Polymers (FRPs) for Reinforcing or Strengthening Concrete Structures.

[43] Alraie A., Sahoo D.R., Matsagar V. (2021). Development of Optimal Anchor for Basalt Fiber–Reinforced Polymer Rods. J. Compos. Constr..

[44] (2020). Standard Practice for Plastics: Dynamic Mechanical Properties: Determination and Report of Procedures.

